# A Novel Strategy for Detecting Recent Horizontal Gene Transfer and Its Application to *Rhizobium* Strains

**DOI:** 10.3389/fmicb.2018.00973

**Published:** 2018-05-15

**Authors:** Xiangchen Li, Wenjun Tong, Lina Wang, Siddiq Ur. Rahman, Gehong Wei, Shiheng Tao

**Affiliations:** ^1^College of Life Sciences and State Key Laboratory of Crop Stress Biology in Arid Areas, Northwest A&F University, Yangling, China; ^2^Bioinformatics Center, Northwest A&F University, Yangling, China

**Keywords:** horizontal gene transfer, sequence similarity, expectation-maximization algorithm, *Rhizobium*, plasmid

## Abstract

Recent horizontal gene transfer (HGT) is crucial for enabling microbes to rapidly adapt to their novel environments without relying upon rare beneficial mutations that arise spontaneously. For several years now, computational approaches have been developed to detect HGT, but they typically lack the sensitivity and ability to detect recent HGT events. Here we introduce a novel strategy, named *RecentHGT*. The number of genes undergoing recent HGT between two bacterial genomes was estimated by a new algorithm derived from the expectation-maximization algorithm and is based on the theoretical sequence-similarity distribution of orthologous genes. We tested the proposed strategy by applying it to a set of 10 *Rhizobium* genomes, and detected several large-scale recent HGT events. We also found that our strategy was more sensitive than other available HGT detection methods. These HGT events were mainly mediated by symbiotic plasmids. Our new strategy can provide clear evidence of recent HGT events and thus it brings us closer to the goal of detecting these potentially adaptive evolution processes in rhizobia as well as pathogens.

## Introduction

Horizontal gene transfer (HGT), also known as lateral gene transfer, is a major process contributing to the evolution of microbes (Soucy et al., 2015). This process can be mediated by the integration of viruses (bacteriophages), transposable elements, or integrative plasmids, often via non-homologous recombination (Soucy et al., 2015). Many HGT events result in genes being transferred from the donor genome to the recipient genome, thereby shaping the latter's evolution in terms of both its functional repertoires and architecture (Jain, 2003; Pál et al., 2005; Treangen and Rocha, 2011; Wiedenbeck and Cohan, 2011). Moreover, previous studies have suggested that the HGT events occur frequently among congeneric species and their strains (Cheeseman et al., 2014; Vos et al., 2015; Remigi et al., 2016; Ruzzini and Clardy, 2016).

Compared with ancient HGT events, recent ones are of paramount importance for the uptake of ready-made genes or operons from the “mobile gene pool,” thus facilitating rapid adaptation to novel environments without the reliance upon rare beneficial mutations arising spontaneously in the population (Treangen and Rocha, 2011). From a clinical perspective, the effectiveness of the HGT is acutely demonstrated by the rapid global spread of antibiotic resistance throughout many bacterial populations (Donnenberg, 2000; Pallen and Wren, 2007; von Wintersdorff et al., 2016). From a mutualism perspective, HGT not only increases the competitiveness of recipient strains for host-bacteria mutualisms but also can benefit the host by promoting an abundance of bacterial species with symbiotic capacity (Dunning Hotopp, 2011; Remigi et al., 2016). Therefore, the reliable and rapid inference of such HGT events can shed light on many adaptive evolutionary processes throughout “the web of life” (Jain, 2003; Ravenhall et al., 2015; Soucy et al., 2015).

Currently, there are two prevailing approaches to detect HGT: phylogenetic and parametric (Ravenhall et al., 2015). The former examines the evolutionary histories of those genes involved and identifies conflicting phylogenies (Jeong et al., 2016). It infers gene trees and compares their topologies against that of a reference species tree, taking the topological incongruence as *prima facie* instances of HGT (Charleston and Perkins, 2006; Bansal et al., 2012; Jeong et al., 2016). Such workflows have the advantage of inferring relatively ancient events, but they could be computationally demanding and rely heavily upon accurate inferences of the gene and species trees (Tofigh et al., 2011; Bansal et al., 2015; Szöll si et al., 2015). The parametric approach searches for sections of a genome that significantly differ from the genomic average, such as in guanine-cytosine (GC) content or codon usage (Daubin et al., 2003; Langille and Brinkman, 2009). The parametric approach requires the transferred segment to be of a relatively distant origin, so that enough divergence has accumulated over time to result in distinguishable compositional features (Ravenhall et al., 2015). This approach suffers from the fact that these features within congeneric relatives may be very similar (Wang, 2001; Lawrence and Ochman, 2002). So far, both approaches seem unsuitable for the robust detection of recent HGT events in closely related bacteria. To this day it remains a challenge, though some researchers have attempted to use their own customized techniques to quantify and describe these recent gene acquisition events (Adato et al., 2015).

Sequence conservation can reveal evidence of HGT, especially regarding recent events (D'Hooghe et al., 1995; Eisen, 2000; Cheeseman et al., 2014). For the vertically inherited orthologous genes across the species, the sequence divergences are positively correlated with the phylogenetic distances (Kim et al., 2014). Furthermore, for the ancient HGT, they would take high selection pressures in the recipient genome, and most of them even have been swept away (Baltrus, 2013). While for the recently transferred genes, selection seems ineffective over such a short period (Hao, 2006; Strese et al., 2014; Vos et al., 2015). Our underlying assumption is that the DNA sequences of the recently transferred genes should be much more conserved than the vertically inherited genes in the recipient genome. In other words, if an extremely conserved homologous gene is observed between two strains, we might suspect it underwent a recent HGT event since divergence of the two strains.

The rhizobia-legume symbiosis is considered an important model of mutualistic evolution and an essential component of sustainable agriculture (Udvardi and Poole, 2013). The symbiosis modules in *Rhizobium* strains are often located in a mobile genetic element on large (>0.2 Mb) plasmids, or so-called symbiotic plasmids (pSyms) (González et al., 2010; Remigi et al., 2016). Recent studies consistently suggest there is evidence for HGT of symbiosis genes within the *Rhizobium* species (Brom et al., 2000, 2004; González et al., 2006; Masson-Boivin et al., 2009; Pérez Carrascal et al., 2016). However, the frequency and biological significance of possible HGT events among the *Rhizobium* spp. remains unclear. Here, we introduce a novel strategy, *RecentHGT*, which allows users to infer recent HGT events between two species at the genome level. We applied our method to ten strains of *Rhizobium* genera and compared our results with other HGT detection methods. We further investigated the location and evolutionary features of the recent HGT genes. Our novel strategy and findings shed light on the impacts and complexities of recent gene acquisition events in microbes.

## Materials and methods

### Data sources

All *Rhizobium* complete genomes were downloaded from NCBI microbial genome resources (http://www.ncbi.nlm.nih.gov/genomes/lproks.cgi) (Wang et al., 1999; González, 2003; Silva et al., 2003; Howieson et al., 2005; González et al., 2006, 2010; Reeve et al., 2010; Acosta et al., 2011; Rogel et al., 2014; Terpolilli et al., 2014; Pérez Carrascal et al., 2016). The sequences were annotated automatically by using the RAST annotation system v2.0 (https://rast.nmpdr.org/) (Overbeek et al., 2014). The annotated genomes of two cheese-associated bacteria were download from Zenodo website (https://zenodo.org).

### Phylogenetic distance

Average nucleotide identity (ANIm) analyses were performed by using pyani.py (https://github.com/widdowquinn/pyani). Briefly, the nucleotide sequences were extracted from the corresponding GenBank files with BioPython (http://biopython.org/) and subsequently used to run pyani (https://github.com/widdowquinn/pyani) in ANIm mode (it uses MUMmer and NUCmer) to align the input genome sequences.

### Pan-genome construction

To identify the homologous genes and to construct the pan-genome of all strains, the ITEP pipeline was used for generation and curation of the protein families (Benedict et al., 2014). The homolog clusters were generated by the Markov Cluster algorithm (with an inflation value of 2.0 and a cutoff value of 0.4), and only single-copy genes between each strain pair were extracted.

### *RecentHGT* implementation and visualization

Similarity values of the protein coding sequences were evaluated by global alignment, using the Needleman-Wunsch alignment algorithm, with the Needle tool in EMBOSS package (Rice et al., 2000). Each sequence-similarity distribution was drawn by the “ggplot2” package and fitted by using the “fitdistrplus” package in R software (v3.4), with the maximum goodness-of-fit estimation and the right-tail Anderson-Darling distance (Delignette-Muller and Dutang, 2015). Recent HGT events were predicted following the main idea of the EM algorithm, which alternates between the steps of guessing a probability distribution over completions of missing data given the current model (known as the E-step), and then re-estimating the model parameters by using these completions (known as the M-step) (Do and Batzoglou, 2008). All steps were implemented by in-house R programming language. The “circlize” statistical package for R software was used to visualize all the inferred recent HGT numbers. Enrichment analysis and data processing were performed with both R and Python software.

### Simulation framework

Two duplicated genomes, with total of 5,000 orthologous protein-coding sequences averaging 951 bp in length, were randomly generated. The mutation rate among the protein loci was suggested to follow the gamma distribution, and the approximately generic magnitude of the per generation spontaneous mutation rate per gene was 10^7^ (NEi et al., 1976; Chen and Zhang, 2013). Here, the mutation rate (μ × 10^7^) of each gene was sampled separately and randomly from a gamma distribution. Then, each of the two genomes was set to evolve independently and continuously. At *i* generation, the mutation probability of *j* gene θ_*ij*_ ranging between 0 and 1 was randomly sampled. If $\theta_{ij}\;<\;\mu_{ij}\;\times \;10^{-7}$, the mutation was set to happen. Then a random site of the sequence was mutated. The maximum generation number was 500 million generations (MG) to represent the relative divergence time of the two genomes. The HGT events were set to occur at four time-points: 50, 10, 5, and 1 MG generations. A single HGT event was simulated by copying 200 genes from the donor to the recipient. The comparative genome analysis was performed as described above. The simulation framework was processed with homemade scripts in Python v3.5.

Next, to validate the performance of our new model, we simulated 12 different HGT events (1, 5, 10, 20, 30, 40, 50, 60, 70, 80, 90, and 100 MG) between two genomes diverged 500 MG with 4,500 orthologous genes. Each HGT event simulated the transfer of 250 genes in total. The ANI value of each of the two diverged genomes was nearly 89.0% similar to the value of *R. phaseoli* N771 and *R. etli* CFN42. Every simulation was repeated 100 times. All the simulation data were then adopted to predict the recent HGT number with five different thresholds: 97.0, 97.5, 98.0, 98.5, and 99.0%. Lastly, each prediction result was divided by the real HGT number to express the prediction sensitivity.

### HGT detection by using phylogenetic and parametric methods

To compare our method with state-of-the-art HGT prediction methods, we picked two core genes, *fixC* and *repB*, presenting in all selected strains and detected as being putative recently transferred among the strains nodulating *P. vulgaris*. Both two genes performed important functions for the pSyms*. fixC* is required for the formation of a functional nitrogenase Fe protein and is involved in nitrogen fixation (Fischer, 1994). The symbiosis genes have been shown to be frequently horizontally transferred (Remigi et al., 2016). *repB* is one of the *repABC* operons which are responsible for the replication and segregation of the plasmids (Cevallos et al., 2008). The frequent HGT of the *repABC* operon has been reported (Castillo-Ramírez et al., 2009). To build the phylogeny of 10 *Rhizobium* strains, every genome was separated into two parts. One was the pSym and the other was the remains of the whole genome. Single-copy core genes among second part were aligned with MAFFT v7.271 (Katoh and Standley, 2013). These alignments were concatenated to infer the maximum likelihood phylogenies with RAxML v8.2.4 with 500 bootstraps, under the GTR model and a gamma correction (GAMMA) for variable evolutionary rates (Stamatakis, 2014). RANGER-DTL-Fast (v2.0) was used as the representative of phylogenetic methods to detect the putative HGT events by reconciling two gene trees against the rooted species tree, using two small transfer costs of 1 and 0.5 instead of the default transfer cost of 3 to make higher HGT probability (Bansal et al., 2012). The species tree and the transfers were created by using iTOL online service (Letunic and Bork, 2016). The highly conserved homologous genes were separated into two parts: one consisted of the chromosomal genes while the other of plasmid genes. We used the BioPython package to calculate the GC content of each gene in each part and compared the two sample means by using the Mann-Whitney-Wilcoxon test (in R software).

## Results

In this section, we introduce a series of processes. These include the initial findings from a pre-experiment, a simulation study of recent HGT events, and the algorithmic design of our novel approach. Next, we tested the approach on 10 *Rhizobium* genomes.

## Pre-experiment

Originally, we had planned to investigate the recently acquired genes in *Rhizobium phaseoli* N771. *R. phaseoli* N771 was isolated from the *Phaseolus vulgaris* root nodule, whose complete genome was recently published. The two query strains, *Rhizobium etli* CFN42^T^ and *Rhizobium etli* Mim1, were earlier defined as the same species but isolated from *P. vulgaris* and *Mimosa affinis* root nodules, respectively (Supplementary Table S1). We measured the genetic relationship between *R. etli* CFN42 and *R. phaseoli* N771, *R. etli* CFN42 and *R. etli* Mim1, and *R. phaseoli* N771 and *R. etli* Mim1 using the average nucleotide identity (ANI). Their ANI values were 90.6, 98.6, and 89.9%, respectively. These values indicated that *R. etli* Mim1 and *R. etli* CFN42 were identified as the same species yet closely related to another species, *R. phaseoli* N771.

**Figure 1:**
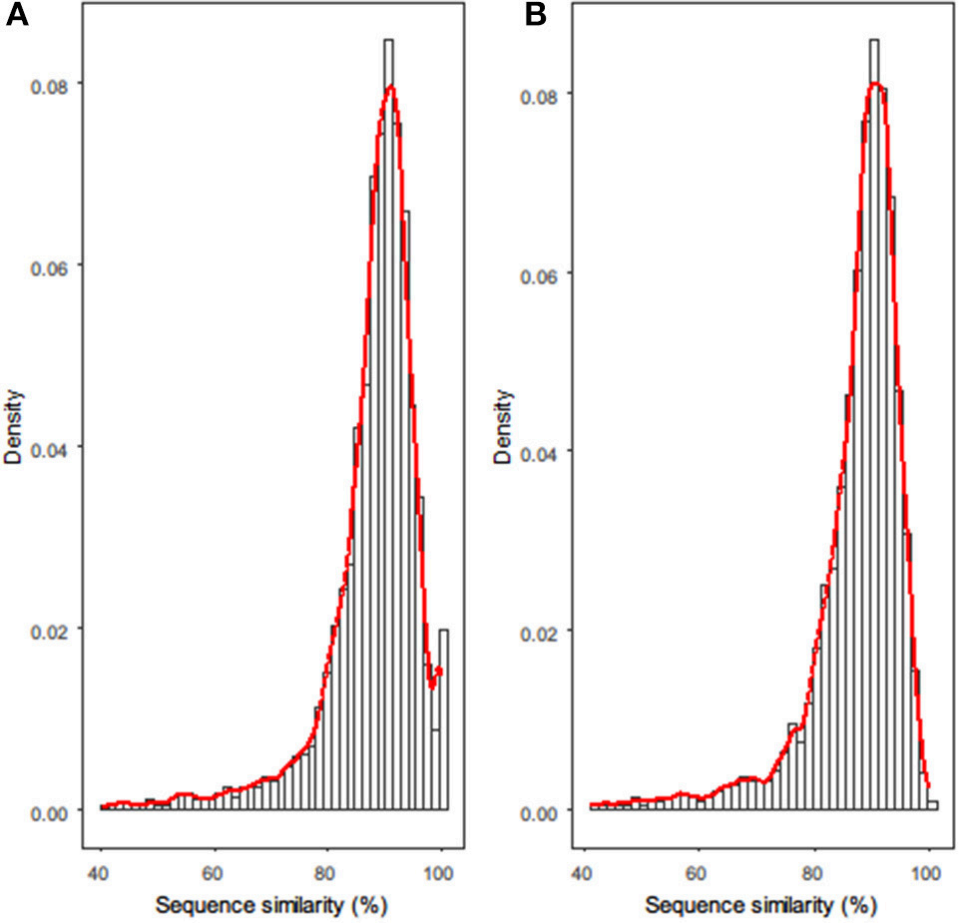
Histograms for sequence-similarity datasets of **(A)** *R. phaseoli* N771 and *R. etli* CFN42, and **(B)** *R. phaseoli* N771 and *R. etli* Mim1. The *x*-axis and *y*-axis in each histogram represent, respectively, the pairwise sequence alignment similarity values of all homologous genes and their densities. The red smoother line drawn over each histogram is the density curve.

Next, all homologous gene families between each strain pair were clustered and pairwise aligned. Histograms of the sequence-similarity values for the homologous genes between *R. phaseoli* N771 and *R. etli* CFN42 are shown in Figure 1A. The empirical distribution appears almost continuous, except for an obvious deviation in the highly conserved interval (HCI) ranging from 98.5 to 100%. Based on the above assumptions for recent HGT, we assumed that most of the homologous genes in this interval did not comply with the theoretical distribution because they had been recently transferred between the two strains.

Additionally, we compared the functions and genomic locations of the highly conserved/similar homologs between two strain pairs (Figure 1). An abnormal HCI was observed in the sequence-similarity distribution of *R. phaseoli* N771 and *R. etli* CFN42 (Figure 1A), and there were totally 172 (3.8%) highly conserved homologs found in the HCI (Supplementary Table S2). Among them, 147 genes located on plasmids (Fisher's exact test; *P* < 0.001), which included many mobile elements and genes involved in nodulation, infection, and nitrogen fixation. The transfers of both groups of genes were frequently reported (Frost et al., 2005; Remigi et al., 2016). The sequence-similarity distribution of *R. phaseoli* N771 and *R. etli* Mim1 more closely approximated a continuous distribution, and only 30 (0.7%) homologs were found in the HCI (Figure 1B, Supplementary Table S3). Among them, we only detected 14 plasmid genes, which is obviously less than that in the HCI of *R. phaseoli* N771 and *R. etli* CFN42. As no coincidence, some ribosomal protein coding genes (15 vs. 13) contributed most to the remaining homologs in the HCIs of both strain pairs. Based on the two different conservation patterns of some plasmids genes, we could speculate that they were recently transferred between *R. phaseoli* N771 and *R. etli* CFN42. In addition, *R. etli* Mim1 qualified to serve as a negative control for the comparison with *R. phaseoli* N771 in recent HGT detection.

## HGT simulation study

**Figure 2:**
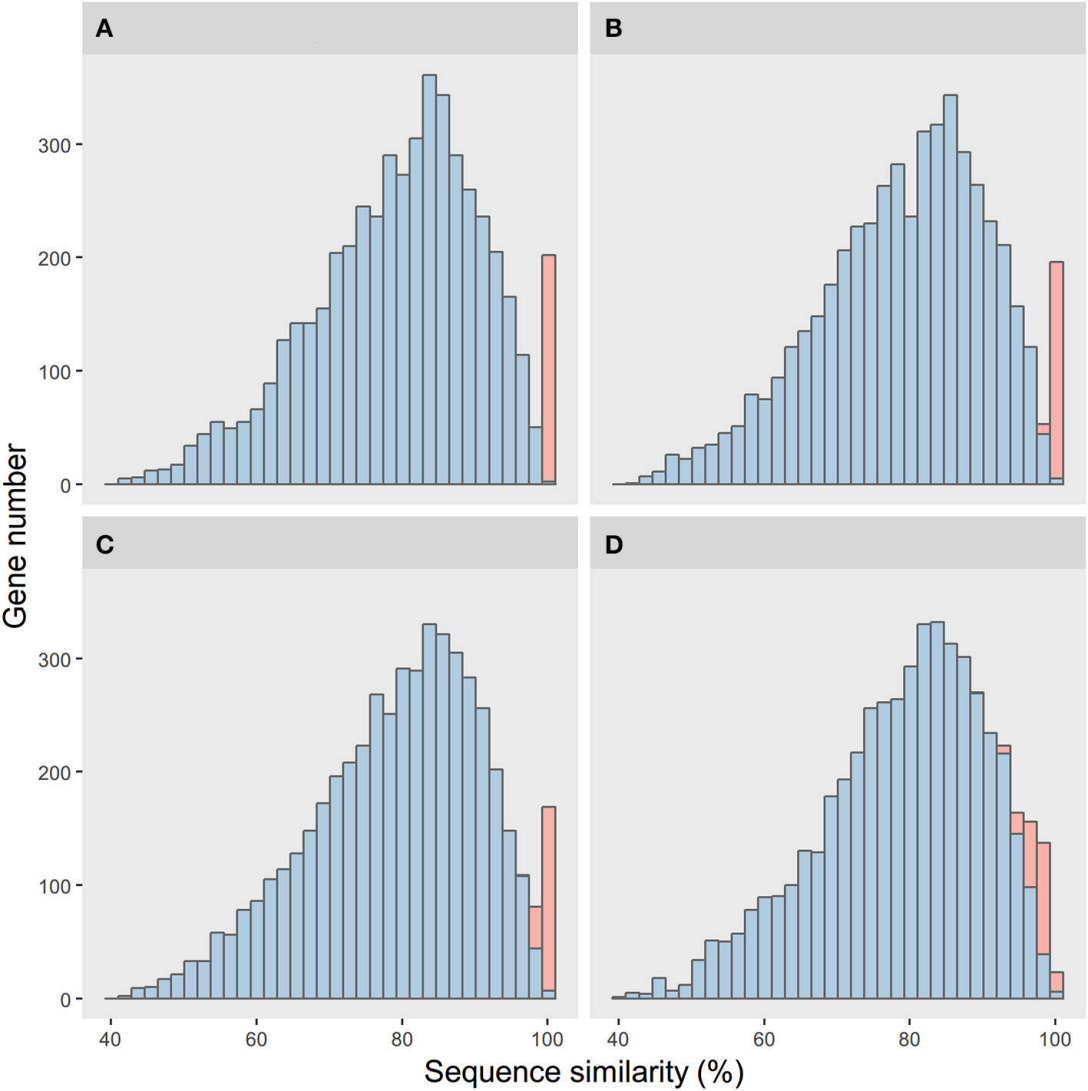
Simulation results of the impacts of different horizontal gene transfer (HGT) histories on the sequence-similarity distribution between two bacterial genomes. The sequence-similarity distribution of the orthologous genes and the HGT genes are in blue and red, respectively. Shown are four time-points for the HGT events, from the near to distant past: **(A)** 1, **(B)** 5, **(C)** 10, and **(D)** 50*MG*.

We conducted a simulation framework to verify whether the unusual amount of highly conserved homologous genes between *R. phaseoli* N771 and *R. etli* CFN42 were indeed the outcome of recent HGT events. Here, two bacterial genomes were generated and set to evolve separately and independently. Let one be the donor and the other be the recipient. The spontaneous mutation rates of different homologous genes were sampled from a gamma distribution. The relative divergence time of the two genomes was set to 500 million generations (MG). We simulated the HGT events at four different time-points to show their impacts on the sequence-similarity distribution between the two bacterial genomes (more details in Materials and; Figure 2).

Every simulated distribution of the vertically transferred genes was similar to that of *R. phaseoli* N771 and *R. etli* Mim1. However, for the HGT events at the different time-points, their sequence conservations were not alike. When the genes had transferred recently, their sequence conservations were observed (Figures 2A–C). These sequence-similarity distributions were similar to the one of *R. phaseoli* N771 and *R. etli* CFN42. However, for the more ancient transfer events, the HGT genes were too divergent to be distinguished from the other orthologous genes (Figure 2D).

## Sequence-similarity distribution fitting

Although most of the highly conserved homologous genes between *R. phaseoli* N771 and *R. etli* CFN42 were recently transferred, some of them, such as the ribosomal proteins and housekeeping genes, were evolutionarily conserved. Therefore, we cannot arbitrarily regard all of these genes between the two bacteria species as being recently transferred genes. Hence, it is necessary to exploit a suitable method to accurately estimate the number of recent HGT genes.

Based on the simulation results, the empirical distribution of *R. phaseoli* N771 and *R. etli* Mim1 was chosen to serve as the null distribution, to minimize the adverse effects of recent HGT events in the distribution fitting. Next, it was necessary to select promising candidates among a predefined set of theoretical distributions to achieve reliable predictions.

**Figure 3:**
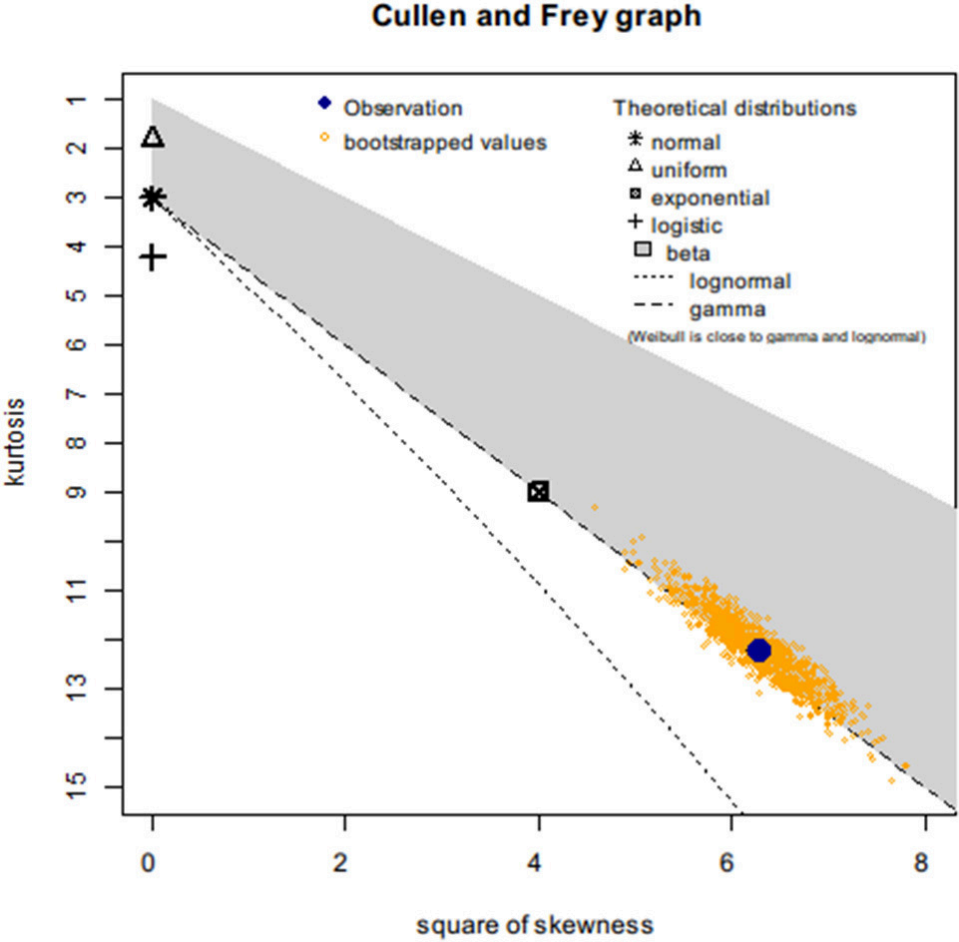
Cullen-Frey graph for the sequence-similarity dataset of *R. phaseoli* N771 and *R. etli* Mim1. Cullen-Frey graph (kurtosis versus square of skewness) for which the recidivism status is *R* = 0. The dataset (observation), indicated in large blue circle. This location is compared to the theoretical locations for various standard distributions. For some distributions (i.e., normal, uniform, logistic, exponential) there is only one possible value for the skewness and kurtosis (for a normal distribution, for example, skewness = 0 and kurtosis = 3), so each given distribution is represented by a single point on the plot. For other distributions, areas of possible values are represented, consisting of lines (gamma and lognormal distributions) or larger areas (beta distribution). Here, the lognormal, the gamma, and the Weibull distributions appeared as potential candidates to fit the sequence-similarity dataset. To evaluate the robustness of the proposed fits, 1,000 non-parametric bootstrap samples were used.

**Figure 4:**
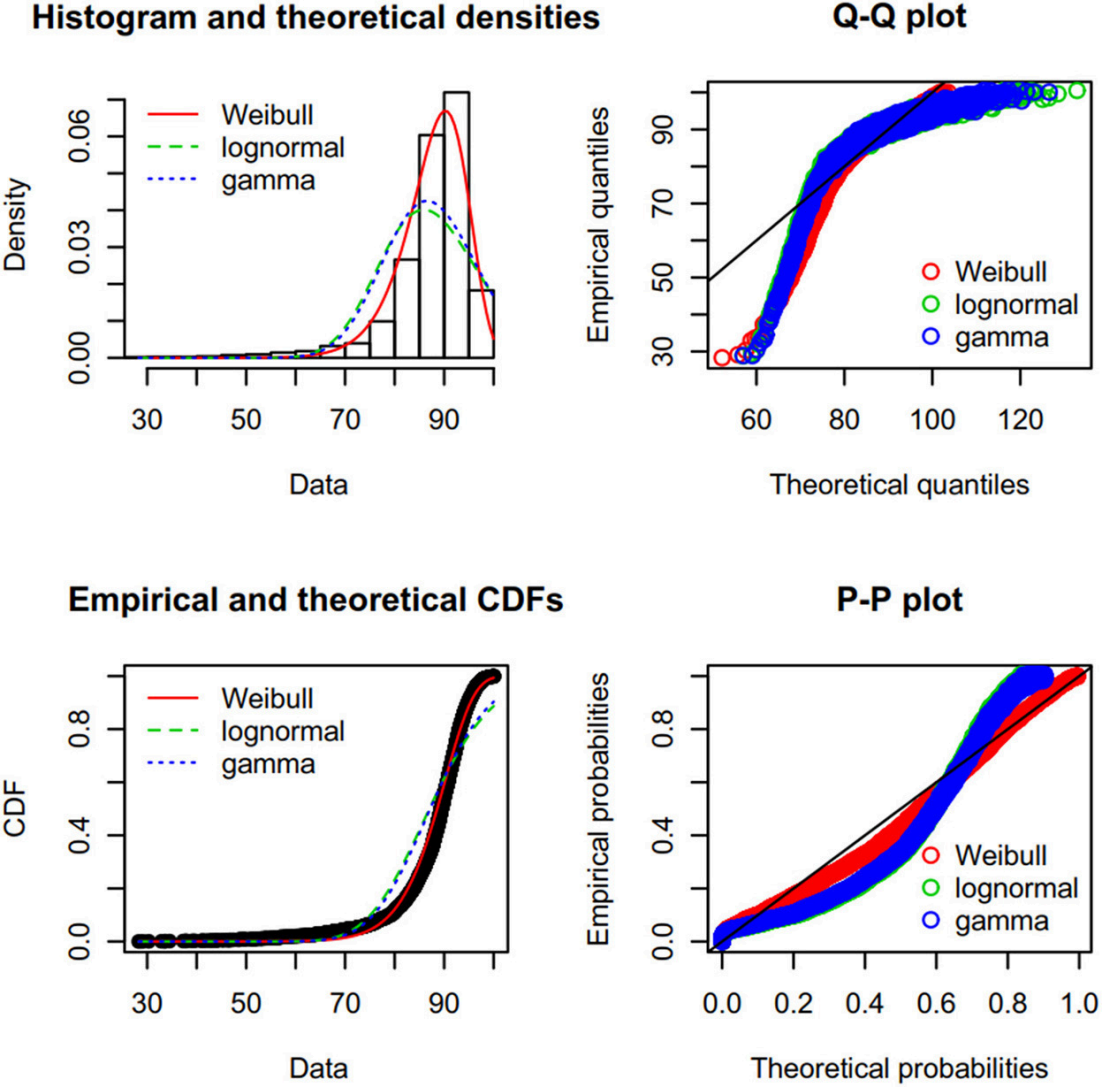
Results from fitting a Weibull distribution to the sequence-similarity dataset of *R. phaseoli* N771 and *R. etli* Mim1. Clockwise: (i) histogram of the empirical distribution (data) superimposed with the density function of three theoretical fitted distributions; (ii) Q-Q plot, a plot of the quantiles of the theoretical fitted distribution (*x*-axis) against the empirical quantiles of the data (*y*-axis); (iii) P-P plot, for each value of the dataset, the cumulative density function of the fitted distribution (*x*-axis) is plotted against the empirical cumulative density function (*y*-axis); (iv) empirical density function of the data superimposed with the cumulative density function of the theoretical fitted distribution. Here, the Weibull distribution (red) performed better than either the gamma (blue) or the lognormal (green) distribution.

First, we created a Cullen-Frey graph with 1,000 bootstraps (Figure 3). In this plot, the values for some common distributions are displayed, in order to help the selection of theoretical distributions to fit the sequence-similarity dataset. The fittings of three common right-skewed distributions were considered: the Weibull, gamma, and lognormal. Then, we used these candidate theoretical distributions to fit the similarity data via maximum goodness-of-fit estimation by employing a right-tail Anderson-Darling distance. Four classical goodness-of-fit plots (i.e., density plot, CDF plot, Q-Q plot, and P-P plot) are shown in Figure 4. According to these plots, the Weibull distribution performed much better than the other two given its better description of the right skewness of the empirical distribution. This skewness is particularly important in the context of a substantial genetic relationship between *R. phaseoli* N771 and *R. etli* Mim1. In addition, we used the Akaike Information Criterion (AIC) and likelihood to measure the relative quality of the three candidate models for the fitted similarity values (Table 1). The results suggested that the Weibull distribution is the preferred model, having the maximal likelihood and the minimum AIC value. Importantly, the Weibull distribution retrieved the lowest error rate than the others at the HCI (Supplementary Table S4). Hence, the Weibull distribution was deemed the most optimal model for the set of sequence-similarity values. The probability density function of a two-parameter Weibull random variable takes this form:

(1) $$f\left(x;\lambda ,\kappa \right)=\left\{ \begin{array}{l} \frac{\kappa }{\lambda }{\left(\frac{x}{\lambda }\right)}^{\kappa -1}e^{-{\left(\frac{x}{\lambda }\right)}^{\kappa }},\\ \;x\geq 0\\ \;0,x<0 \end{array}\right.$$

where κ > 0 is the shape parameter, and λ > 0 is the scale parameter.

**Table 1:** Numerical results of fitting the sequence-similarity distribution between *R. phaseoli* N771 and *R. etli* Mimi1 by different candidate distributions.

**Measures**	**Weibull**	**Lognormal**	**Gamma**
Likelihood	−14,208.89	−15,654.55	−15,449.04
Akaike Information Criterion	27,221.43	38,480.93	36,302.79

## An expectation-maximization algorithm for predicting recent HGT

It was possible to accurately predict the number of genes not belonging to the theoretical sequence-similarity distribution. Here, we followed the main idea of the expectation-maximization (EM) algorithm. Let *G*_1_ and *G*_2_ be two given genomes, and *S* be the sequence-similarity values set of all homologous genes, θ be the estimated parameter of the Weibull distribution, and ρ be the threshold for the recent HGT genes. The *S* is split into two sets: the general continuous interval *S*_*c*_ ∈ [40%, ρ], as the training set includes most of the vertically transferred genes, and the query interval *S*_*h*_ ∈ [ρ, 100%], as the testing set includes most of the potential recent HGT genes. The details of an EM algorithm for detecting HGT are given below:

- The “E-step” for EM algorithm here can be understood by using the Weibull model to fit the *S*_*c*_, estimating the preliminary θ_*t* = 0_, and predicting the number *N*_*t* = 0_ of genes in the HCI belonging to the theoretical distribution.
- For the “M-step,” we randomly picked *N*_*t* = 0_ genes from the *S*_*h*_, and added them into the *S*_*c*_. Then we estimated θ_*t* = 1_ by fitting the new *S*_*c*_, predicted *N*_*t* = 1_, and repeated these steps no more than 1,000 times. The repetition stopped when the convergence is met for *N*_*t*+1_ = *N*_*t*_. Let the last estimation be *N*_*final*_. In this way, the predicted number of recent HGT genes was obtained as *X* = *S*_*h*_ − *N*_*final*_.

**Figure 5:**
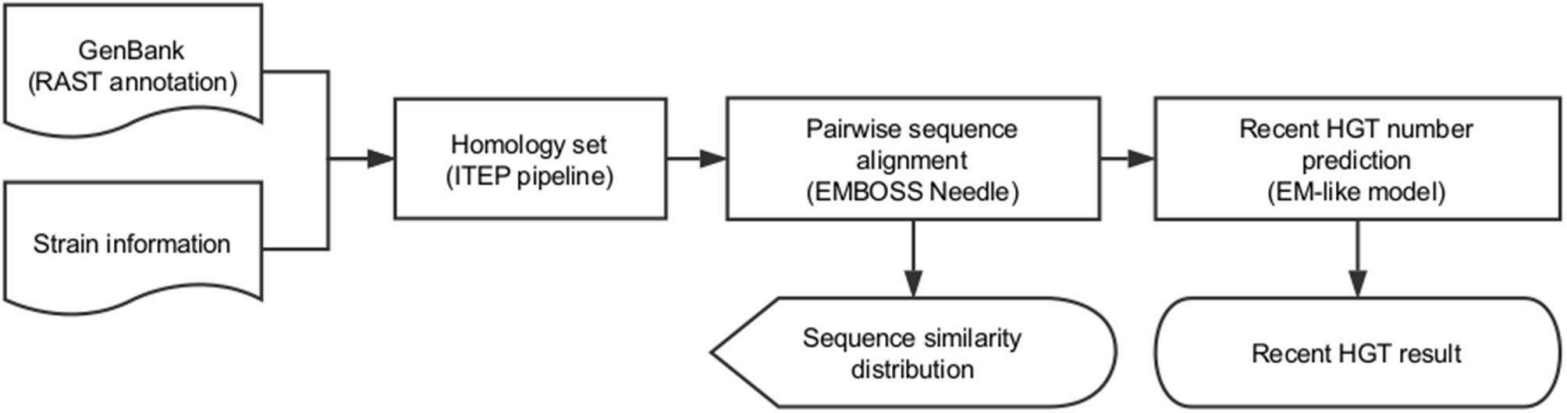
Workflow of the *RecentHGT* analysis pipeline for detection of recent horizontal gene transfer (HGT).

We implemented this EM model in the R programming language, wrapped by Python programming language to complete all above steps automatically, and named it *RecentHGT* (Figure 5).

## Threshold choice and model validation

**Figure 6:**
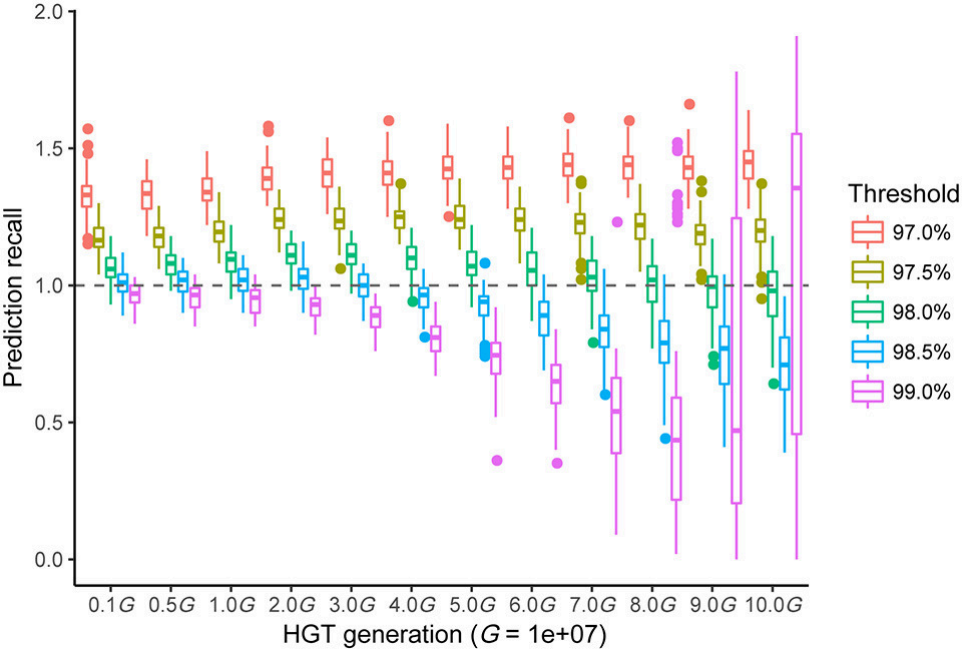
All prediction performances with different sequence-similarity thresholds of the different simulated horizontal gene transfer (HGT) events between two bacterial genomes. The horizontal axis shows the 12 HGT events (1-10 *MG*). Each boxplot denotes the prediction recalls of 100 repeated simulations with a given threshold for the *RecentHGT*. The vertical axis represents the prediction recalls and the perfect recall rate of 1.0 is denoted by the dotted line.

It was necessary to validate our new method and to choose an optimal threshold to achieve the best performance. Here, we simulated two independently evolved bacterial genomes separated by a common ancestor that had different transfer histories (see Materials and Methods). The ANI value of each genome was similar to that of *R. phaseoli* N771 and *R. etli* CFN42. The prediction recall rate—the predicted HGT number divided by real HGT number—was calculated by using the *RecentHGT* to predict each data simulation with a given threshold, ρ (Figure 6). The results showed that our new method performed well on the HGT detection. Importantly, the more recently the HGT occurred, the more accurately and reliably was the HGT number estimated. Meanwhile, to ensure robustness, the ρ near 98.5% could be considered as the best choice for the recent HGT prediction.

**Figure 7:**
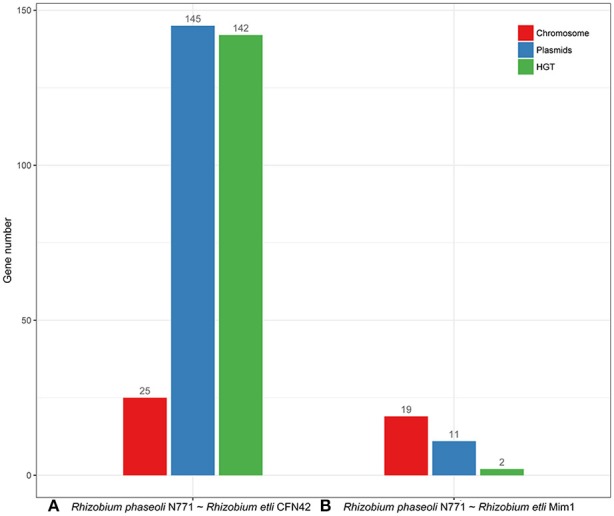
Statistics for the highly conserved homologous genes on the chromosome (red) and plasmids (blue), and the estimated number of recent horizontal gene transfer (HGT) genes (green) between two *Rhizobium* strains. **(A)** *R. phaseoli* N771 and *R. etli* CFN42, and **(B)** *R. phaseoli* N771 and *R. etli* Mim1.

We compared the predicted numbers of HGT genes between *R. phaseoli* N771 and *R. etli* CFN42, as well as between *R. phaseoli* N771 and *R. etli* Mim1 (as the control), with the optimal threshold of ρ = 98.5% (Figure 7). The predicted HGT number was almost entirely consistent with the number of plasmid genes between *R. phaseoli* N771 and *R. etli* CFN42. This indicates that most of the recent HGT genes were plasmid-mediated. Meanwhile, the predicted number of recent HGT genes between *R. phaseoli* N771 and *R. etli* Mim1 is only two. Therefore, although *R. etli* CFN42 and *R. etli* Mim1 belonged to the same species, the recent HGT events were only observed between *R. phaseoli* N771 and *R. etli* CFN42. Hence, our method was able to accurately predict the number of recent HGT genes occurring between two closely related species.

Although, the same prediction for *R. etli* CFN42 and *R. etli* Mim1 was performed, the genetic relationship between these two strains was too close to reliably predict the recent HGT genes, since these were masked by thousands of highly conserved orthologous genes (Supplementary Figure S1). Therefore, in the sections that follow we ignored the HGT detection between two closely related strains (ANI > 95%).

## Analysis of 10 *Rhizobium* genomes

**Figure 8:**
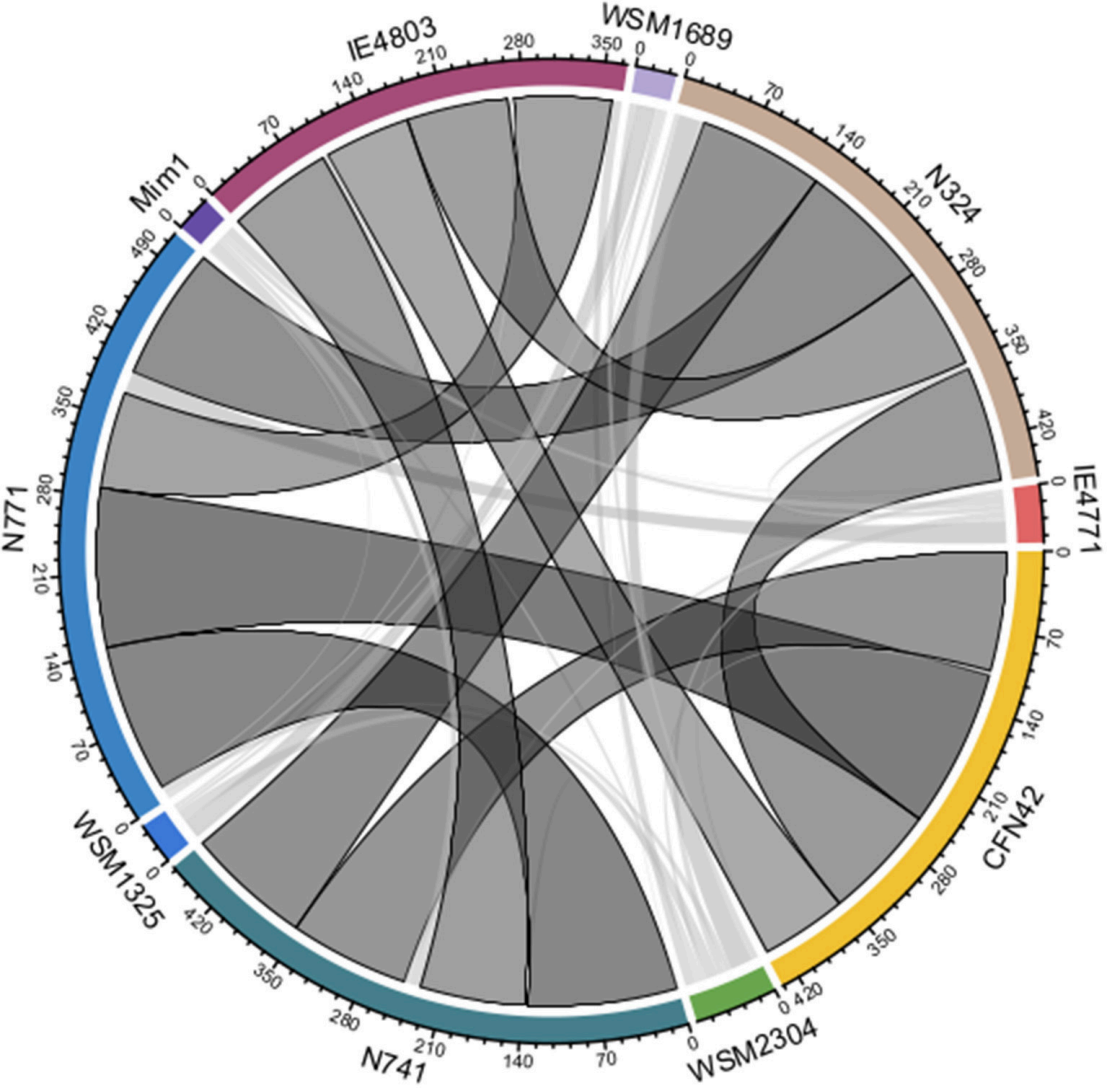
All predicted horizontal gene transfer (HGT) results among the 10 *Rhizobium* strains. The width of a link represents the predicted number of the recent HGT homologous genes between two genospecies (ANI < 95%). The darker the link color is, the larger the predicted magnitude of the recent HGT. The black border indicates the predicted number over 20.

Here, it is nontrivial to consider more *Rhizobium* strains for the extent of recent HGT events, and the role of pSyms in such a process. By doing so, we could test our strategy more fully and rigorously. Ten complete sequences of *Rhizobium* strains isolated from *Phaseolus vulgaris, Mimosa affinis*, and additional *Trifolium* spp. root nodules, were collected (see Supplementary Table S1). The pairwise ANI values of these genome sequences ranged from 87.81 to 98.60% (Supplementary Table S5). Furthermore, we applied the strategy *RecentHGT* to 43 genospecies pairs in total (ANI < 95%), for which their prediction results are shown as a circular layout (Supplementary Table S6; Figure 8). Ten genospecies pairs, or about one-quarter of all pairs, shared the prominent HGT links. All 10 of them were isolated from the *P. vulgaris* root nodules. Conservation of the recent HGT genes in these sequence-similarity distributions was distinct (Supplementary Figure S2). This suggested that the recent HGT events were very frequent within the strains nodulating *P. vulgaris*. However, for the other 33 genospecies pairs, the predicted HGT numbers were generally very few. Thus, HGT events among these strains seem to occur only rarely.

**Figure 9:**
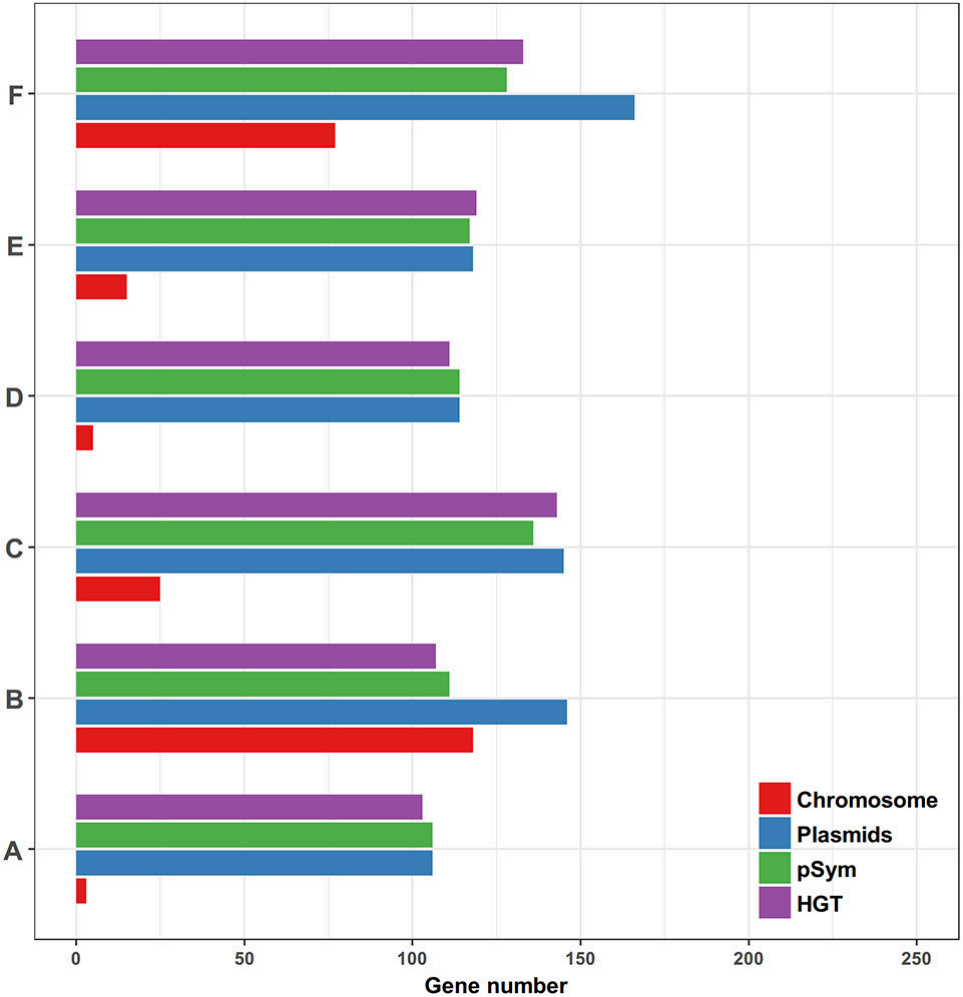
Comparison of the predominant prediction results and the conserved homologous genes locating on different genomic replicons among the six genospecies pairs (ANI < 95%). **(A)** *Rhizobium* sp. N324 and *Rhizobium etli* CFN42, **(B)** *Rhizobium* sp. N741 and *Rhizobium etli* CFN42, **(C)** *Rhizobium phaseoli* N771 and *Rhizobium etli* CFN42, **(D)** *Rhizobium* sp. N324 and *Rhizobium* sp. N741, **(E)** *Rhizobium* sp. N324 and *Rhizobium phaseoli* N771, and **(F)** *Rhizobium* sp. N741 and *Rhizobium phaseoli* N771.

We then compared the prediction results and the real numbers of genes on the chromosomes and plasmids, and the pSyms, respectively (Figure 9). The results showed that the pSyms occupied most of the recent HGT genes. To further demonstrate the impact of the pSym genes upon the empirical distributions, we implemented the same comparative genomics analysis of the 10 genospecies pairs by removing all genes located on the pSyms (Supplementary Figure S3). Notably, these distributions seem to return to the continuous. Both results together indicate the pSym as the key agent of the recent HGT events within *Rhizobium* genera.

To determine whether the predicted numbers of recent HGT genes were associated with the phylogenetic distances of the two *Rhizobium* strains, a correlation analysis between the ANI values and HGT numbers was applied. This analysis showed that they were not significantly correlated (Pearson's correlation coefficient; *r* = −0.412; *P* = 0.16). Therefore, there might exist other external forces that boost HGT, such as the induction of host plant traits.

## Comparison with other methods

**Figure 10:**
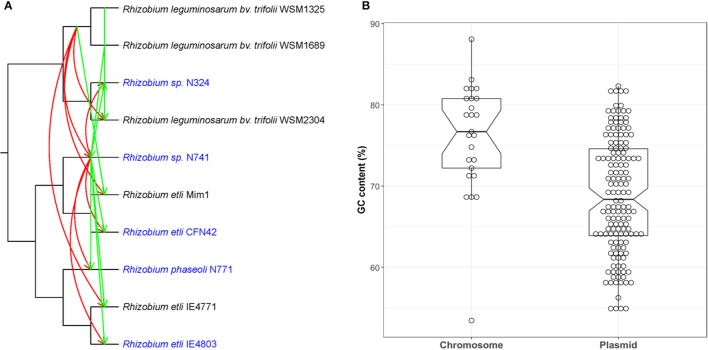
Comparison of *RecentHGT* with both phylogenetic and parametric methods. **(A)** HGT detection of two core genes *fixC* and *repB* using RANGER-DTL with a transfer cost of 0.5. The HGT events predicted by the RANGER-DTL for *fixC* and *repB* are denoted by the red and green lines, respectively. Arrows represent the inferred transfer direction (from donor to recipient). The strains nodulating *Phaseolus vulgaris* recently acquired the two genes predicted by *RecentHGT* were marked as blue; **(B)** the GC contents comparison of highly conserved chromosomal and plasmid genes between *R. phaseoli* N771 and *R. etli* CFN42.

Since phylogenetic approaches are considered the gold standard in HGT detection, we compared our approach with a representative existing HGT method as applied to the same 10 *Rhizobium* genomes. The phylogenetic approaches detect inconsistencies in gene and species evolutionary history. Here, we selected two pSym genes, *fixC* and *repB*, presenting in all selected strains as the test data, recently acquired among five strains nodulating *P. vulgaris* which were predicted by our method. we used RANGER-DTL—a commonly used software—due to its rapidity and accuracy to do a cross-validation. To implement the reconciliation, a species tree based on the concatenated single-copy core genes, and two gene trees based on *fixC* and *repB*, were constructed, respectively. Next, we applied the RANGER-DTL-U algorithm over the three undated trees by using two small transfer costs of 1 and 0.5 (default is 3), respectively. The lower transfer cost, the higher HGT probability. The HGT results from RANGER-DTL with the transfer cost of 0.5 were almost consistent with our method and suggested that *Rhizobium* sp. N741 is the donor of both genes to other four strains nodulating *P. vulgaris* (Figure 10A; Supplementary Table S7). However, the HGT results from RANGER-DTL with the transfer cost of 1 were partly different and insufficient to describe a complete transfer scenario as the above (Supplementary Figure S4; Supplementary Table S7). Notably, RANGER-DTL also retrieved some possible HGT events for the strains nodulating *Mimosa affinis*, and *Trifolium* spp. In spite of this, the recent HGT inference by RANGER-DTL relies on a priori selection of the optimal transfer cost.

Parametric approaches are prevalent in HGT detection and known to be very fast because they do not require comparison with other genomes. We compared the GC contents of the predicted recent HGT genes on the pSyms with other highly conserved chromosomal genes between *R. phaseoli* N771 and *R. etli* CFN42 (Figure 10B). The results showed that the values between HGT and other chromosomal genes were significantly different (two-tailed Mann-Whitney *U*-test, *P* < 0.01). This result supported the identification of the predicted recent HGT genes because they were reported to be usually AT-rich (Hildebrand et al., 2010). However, this approach was not sensitive enough for extracting all recent HGT genes between the two closely related species.

## Application to two distantly related species

In order to evaluate the performance of our method between two distantly related species, we further applied *RecentHGT* to two cheese-associated bacteria, *Brevibacterium antiquum* CNRZ918 and *Corynebacterium casei* LMG S-19264, sharing a large recent HGT cluster (Bonham et al., 2017). *B. antiquum* and *C. casei* belong to different orders, *Micrococcales* and *Corynebacteriales*, respectively. In spite of this, *RecentHGT* successfully predicted very similar number of the recently HGT genes (61 vs. 65) between two species at the practical threshold of ρ = 85% based on their sequence-similarity distribution (Supplementary Table S8; Supplementary Figure S5). Compared to two *Rhizobium* species, their sequence-similarity distribution has an increased negative skewness and a reduced kurtosis indicating their farther phylogenetic distance and fewer homologs (Supplementary Table S9). Therefore, the scenario of *RecentHGT* ought to be suitable for a wide range of bacteria.

## Discussion

Sequence conservation is usually used for detecting HGT, especially for building the “web of life,” and the highly conserved homologs are often regarded as the recent HGT candidates based on the molecular clock hypothesis (Smillie et al., 2011; Yamashita et al., 2014; Corel et al., 2016). However, some false positive candidates need to be removed because some vertically inherited housekeeping genes are also highly conserved. In this study, we developed a novel strategy, *RecentHGT*, by exploiting the Weibull distribution and the EM model to describe the vertically inherited genes and reliably predict the number of recent acquired genes between two bacteria species, respectively. *RecentHGT* uses a pairwise sequence alignment instead of multiple sequence alignment to ensure the reproducibility of the similarity calculation (Busse et al., 2010), and avoids having to infer a reliable species tree and reconciling non-binary gene trees as well as choose optimal costs for duplication, transfer, and loss events (Szöll si et al., 2015; Jacox et al., 2017; Lai et al., 2017). Moreover, we have shown that this method can be applied to two distantly related species by selecting a practical threshold of HCI.

We have successfully implemented *RecentHGT* to 10 *Rhizobium* strains nodulating different legume plants. The recent gene acquisitions were only detected between two species isolated from *Phaseolus vulgaris* root nodules, and mainly mediated by the symbiotic plasmids. This implies that the symbiosis modules of the rhizobial species in this study were host-specific. But such phenomenon may not apply in all cases and need to be further examined. The findings have enhanced our understanding of *Rhizobium*-legume symbiosis.

Our strategy may shed light on the recent gene acquisition among bacteria living in a same biological environment. Detecting such events is important for the study of mutualism as well as for molecular epidemiology (Eisen, 2000). The former field would help us to explore the mechanisms by which recipient genomes evolve to completely exploit the plant niche space accessed by acquired symbiosis genes, and to better understand the selective forces that govern the emergence of symbiotic nitrogen fixation in nature (Remigi et al., 2016). Further, our strategy may enhance the inference of putative horizontally transferred genes adapted from work on the human microbiome and phage-mediated transduction (Smillie et al., 2011; Touchon et al., 2017). The latter field would benefit greatly from a more sensitive reconstruction of the emergence of virulent, often drug-resistant, strains (von Wintersdorff et al., 2016). In future, this method will be applied to additional bacterial genomes and integrated with more state-of-the-art approaches, such as several newly developed phylogenetic technologies, so that cross-validation and accurate tracing of the donors and recipients can be facilitated (Adato et al., 2015; Jacox et al., 2016).

## Conclusion

*RecentHGT* can effectively detect the existence of recent HGT events between two bacterial species at the genome level, while also able to estimate the reliable number of the transferred genes. Besides, our method detected several large-scale recent HGT events among 10 *Rhizobium* strains, which contributed most to the expansion of symbiotic traits.

*RecentHGT* requires pre-annotated genomes—i.e., protein-coding genes should be predicted by using the RAST pipeline prior to running the program. The *RecentHGT* program with its source code, example files (for the 10 *Rhizobium* genomes), simulation scripts, and documents are freely available online (https://github.com/cvn001/RecentHGT). This program can be run on command-line terminals of OS X and Linux computers/servers.

## Author contributions

XL, WT, GW, and ST conceived and designed experiments; LW collected data. XL, WT performed all experiments and analyzed the data. XL and WT drafted the manuscript. SR reviewed the manuscript. All authors read and approved the final manuscript.

### Conflict of interest statement

The authors declare that the research was conducted in the absence of any commercial or financial relationships that could be construed as a potential conflict of interest.

## Supplementary material

Supplementary Figure S1 Histogram for the sequence-similarity dataset of *R. etli* CFN42 and *R. etli* Mim1. ANI, average nucleotide identity.

Supplementary Figure S2 Histograms for the sequence-similarity datasets of 10 *Rhizobium* species pairs with predominant numbers of recent horizontally transferred genes. ANI, average nucleotide identity.

Supplementary Figure S3 Histograms for the sequence-similarity datasets of 10 species pairs with predominant recent horizontal gene transfers excluding all genes on the pSyms. ANI, average nucleotide identity.

Supplementary Figure S4 HGT detection of two genes *fixC* and *repB* by RANGER-DTL with transfer cost of 1. The HGT events predicted by the RANGER-DTL for *fixC* and *repB* are denoted by the red and green lines, respectively. Arrows represent the inferred transfer direction (from donor to recipient). The strains nodulating *Phaseolus vulgaris* that recently acquired the two genes predicted by *RecentHGT* were marked as blue.

Supplementary Figure S5 Histogram for the sequence-similarity datasets of two cheese-associated bacteria, *Brevibacterium antiquum* CNRZ918 and *Corynebacterium casei* LMG S-19264. The red smoother line drawn over the histogram is the density curve.

Supplementary Table S1 The *Rhizobium* strains used in this study.

Supplementary Table S2 Highly conserved homologous genes between *R. phaseoli* N771 and *R. etli* CFN42. The symbiotic genes, the mobile element proteins, and the *repABC* operon are respectively highlighted in yellow, green, and blue; the chromosomal genes are in gray.

Supplementary Table S3 Highly conserved homologous genes between *R. phaseoli* N771 and *R. etli* Mim1. The chromosomal genes are shown in gray.

Supplementary Table S4 Comparison of the fitting precision of three candidate reference distributions, Weibull, gamma and lognormal at the HCI (from 98.5 to 100%).

Supplementary Table S5 Pairwise comparisons of average nucleotide identity among the 10 *Rhizobium* strains. Strains with ANI values less than 95% are suitable for the recent HGT detection.

Supplementary Table S6 All predicted numbers of recently transferred genes between the 36 *Rhizobium* species pairs. The results >20 were considered prominent and these are shown in gray.

Supplementary Table S7 All inferred HGT events from RANGER-DTL by using two different transfer costs of 0.5, 1, respectively.

Supplementary Table S8 (A) Highly conserved homologs between two cheese-associated bacteria, *Brevibacterium antiquum* CNRZ918 and *Corynebacterium casei* LMG S-19264; (B) The reference recent HGT genes cluster between two species.

Supplementary Table S9 Fitted Weibull distribution parameters of sequence-similarity distributions with no HCIs of two species pairs, *Rhizobium phaseoli* N771 and *Rhizobium etli* CFN42, and *Brevibacterium antiquum* CNRZ918 and *Corynebacterium casei* LMG S-19264.
