# Supplementary figures and images for: A Novel Strategy for Detecting Recent Horizontal Gene Transfer and Its Application to Rhizobium Strains
Source: Front Microbiol. 2018 May 15;9:973. doi: 10.3389/fmicb.2018.00973 (PMC5968381; doi:10.3389/fmicb.2018.00973)

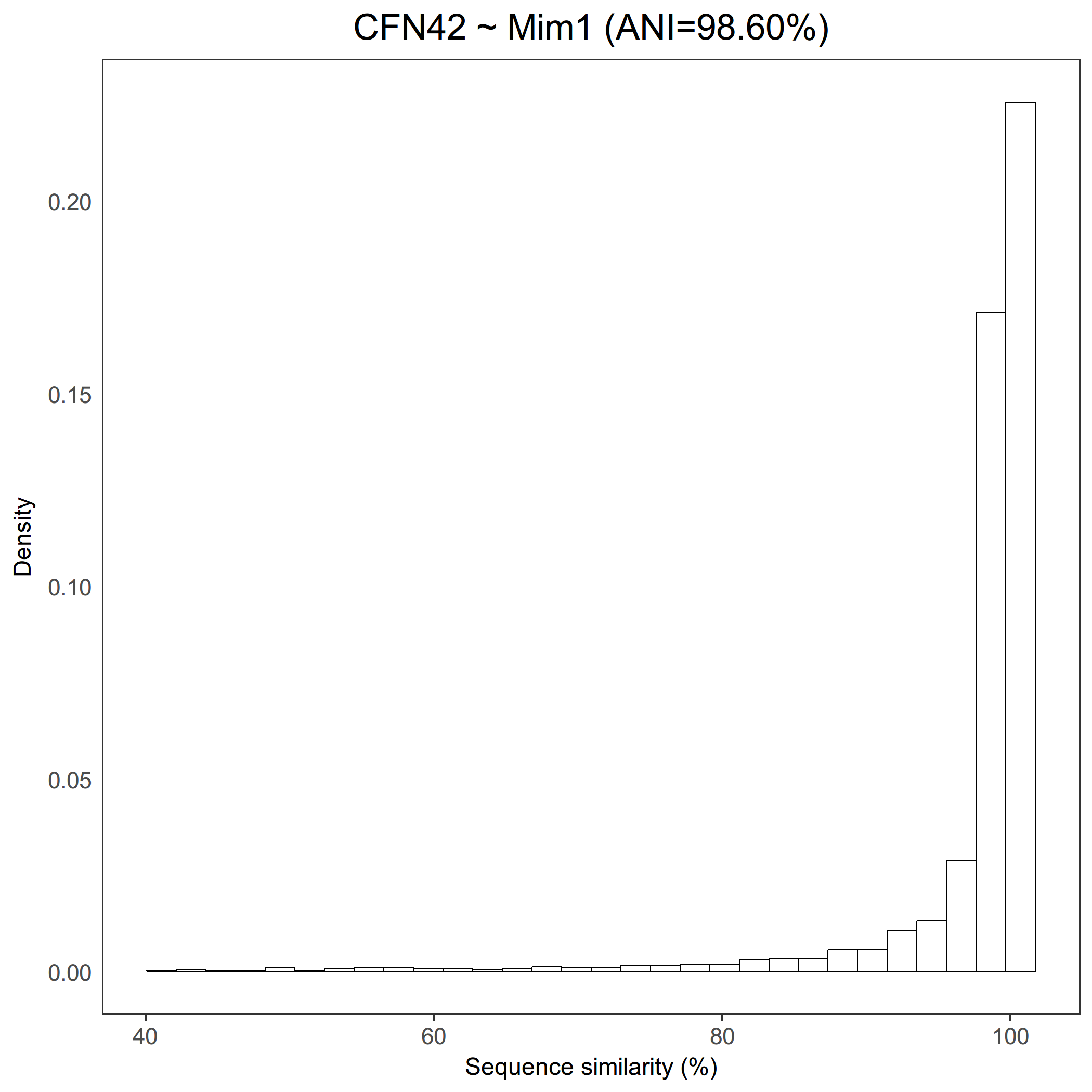

Supplement: Supplementary Figure S1 — Histogram for the sequence-similarity dataset of R. etli CFN42 and R. etli Mim1. ANI, average nucleotide identity. [file Image_1.TIF]

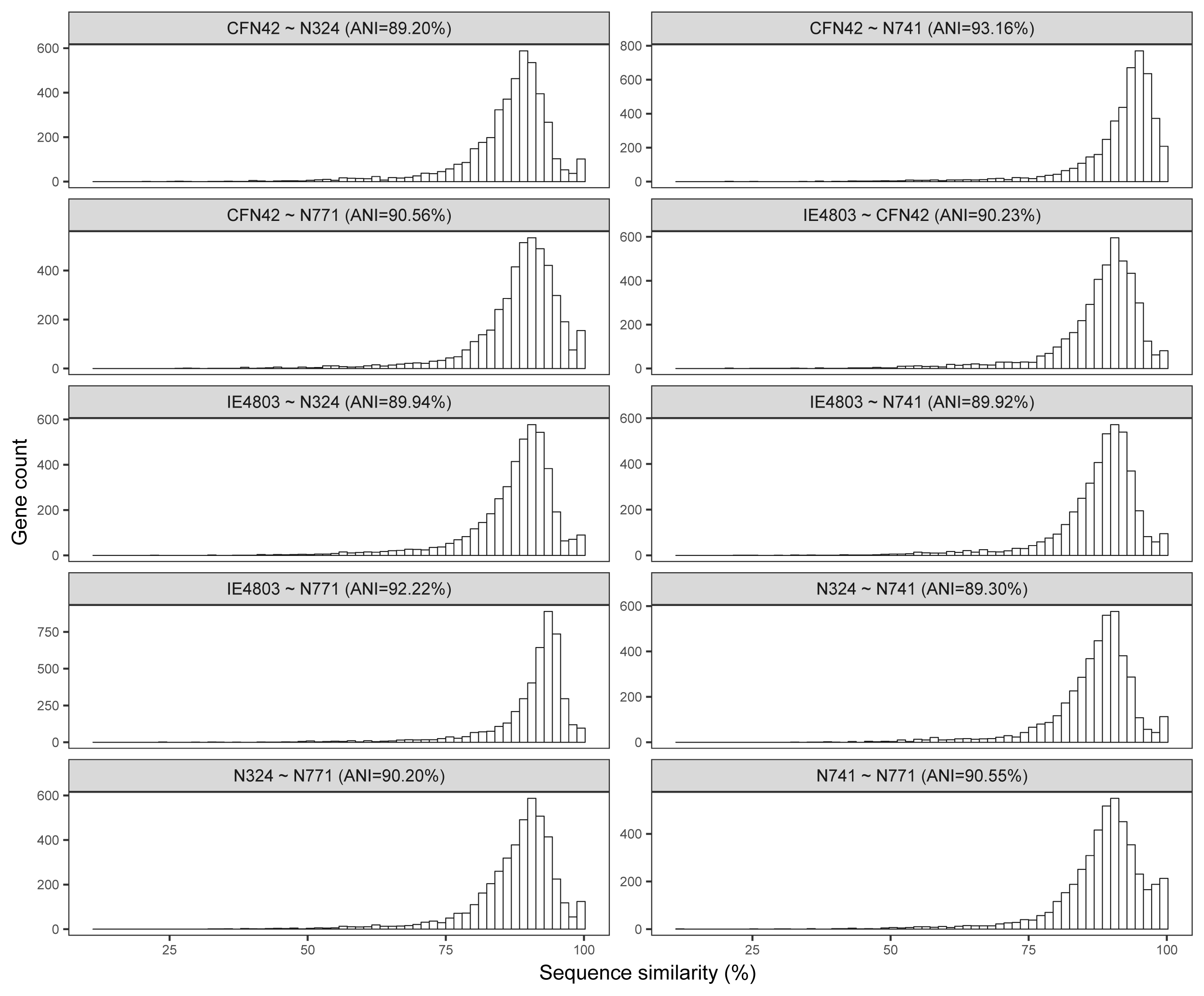

Supplement: Supplementary Figure S2 — Histograms for the sequence-similarity datasets of 10 Rhizobium species pairs with predominant numbers of recent horizontally transferred genes. ANI, average nucleotide identity. [file Image_2.TIF]

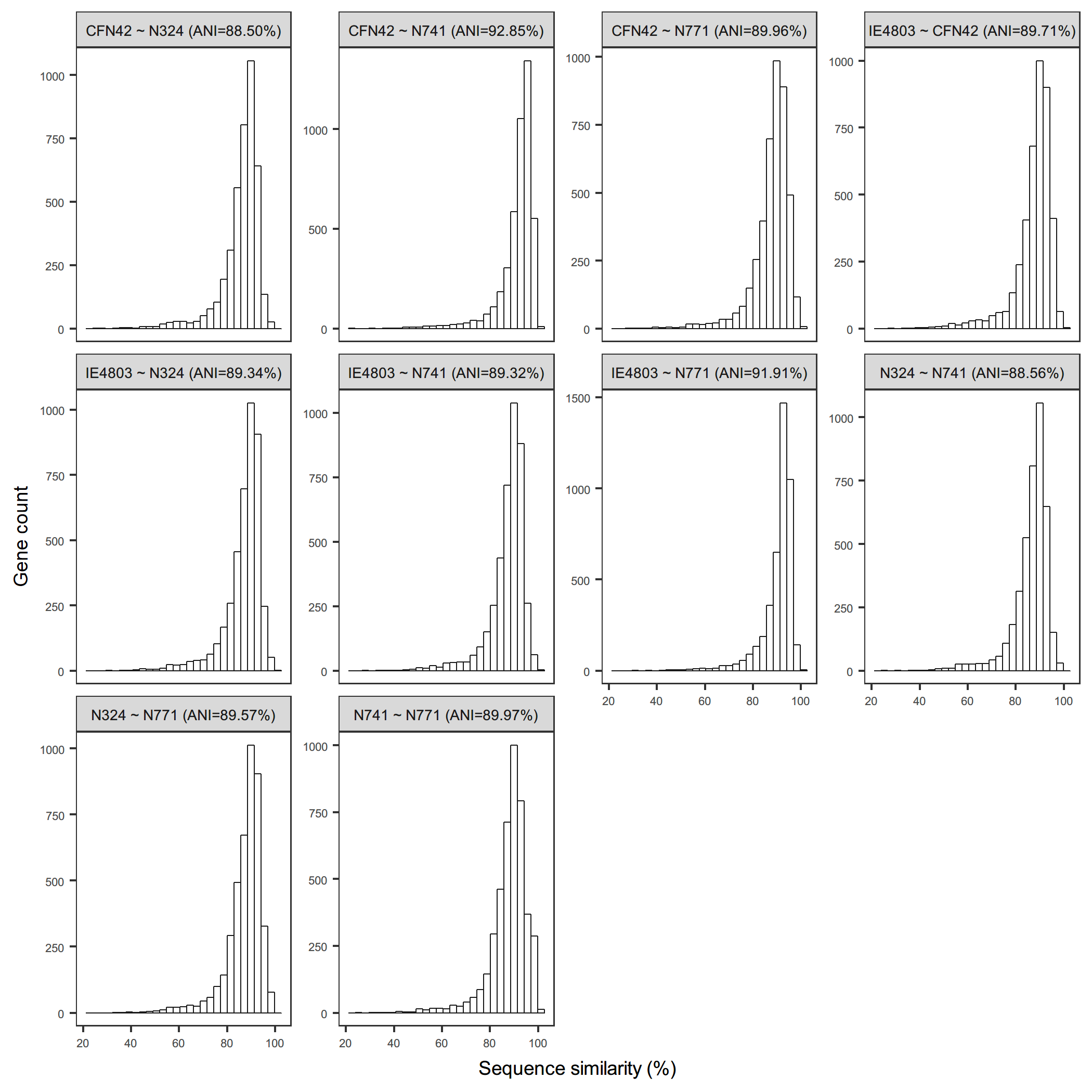

Supplement: Supplementary Figure S3 — Histograms for the sequence-similarity datasets of 10 species pairs with predominant recent horizontal gene transfers excluding all genes on the pSyms. ANI, average nucleotide identity. [file Image_3.TIF]

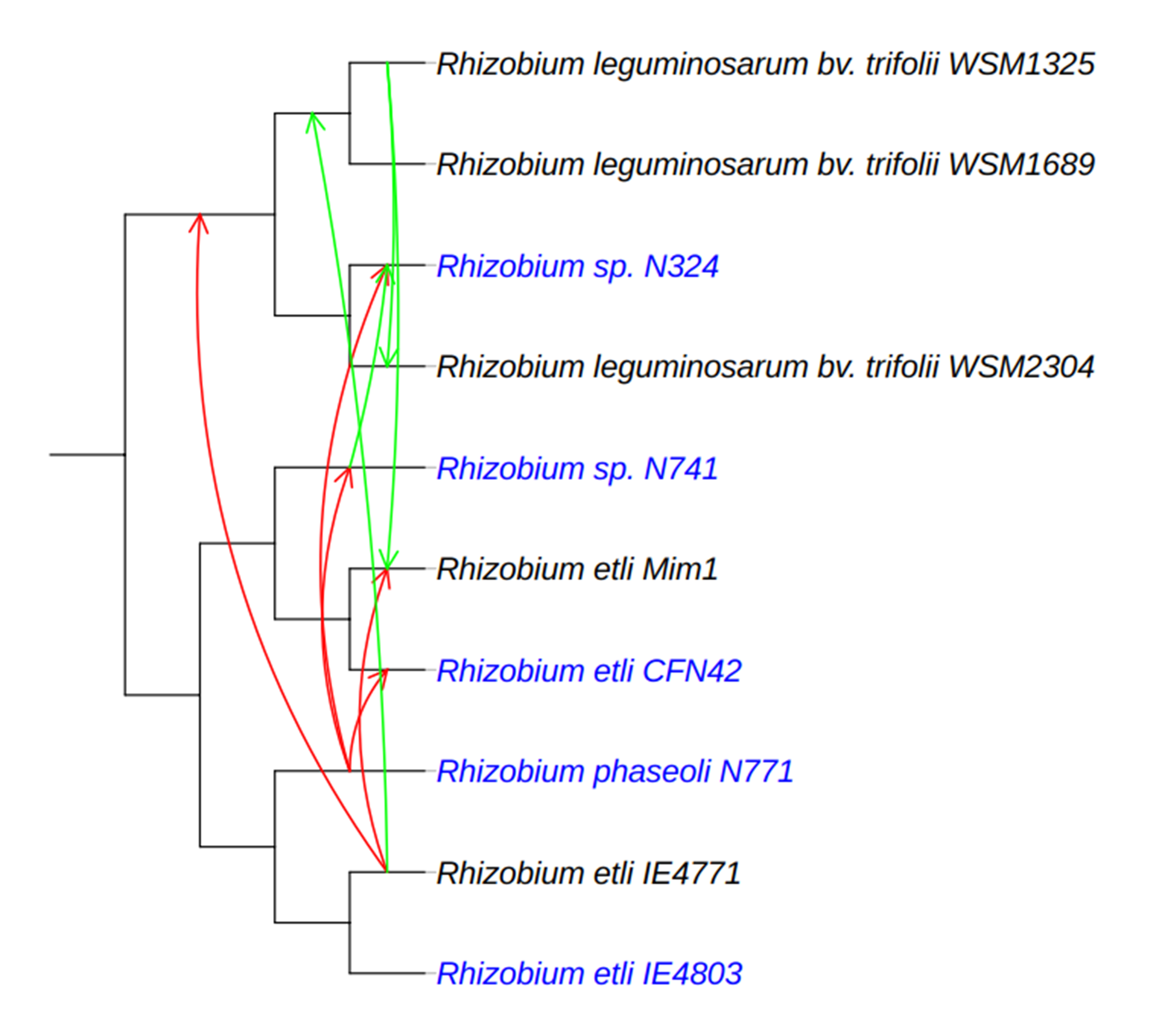

Supplement: Supplementary Figure S4 — HGT detection of two genes fixC and repB by RANGER-DTL with transfer cost of 1. The HGT events predicted by the RANGER-DTL for fixC and repB are denoted by the red and green lines, respectively. Arrows represent the inferred transfer direction (from donor to recipient). The strains nodulating Phaseolus vulgaris that recently acquired the two genes predicted by RecentHGT were marked as blue. [file Image_4.TIF]

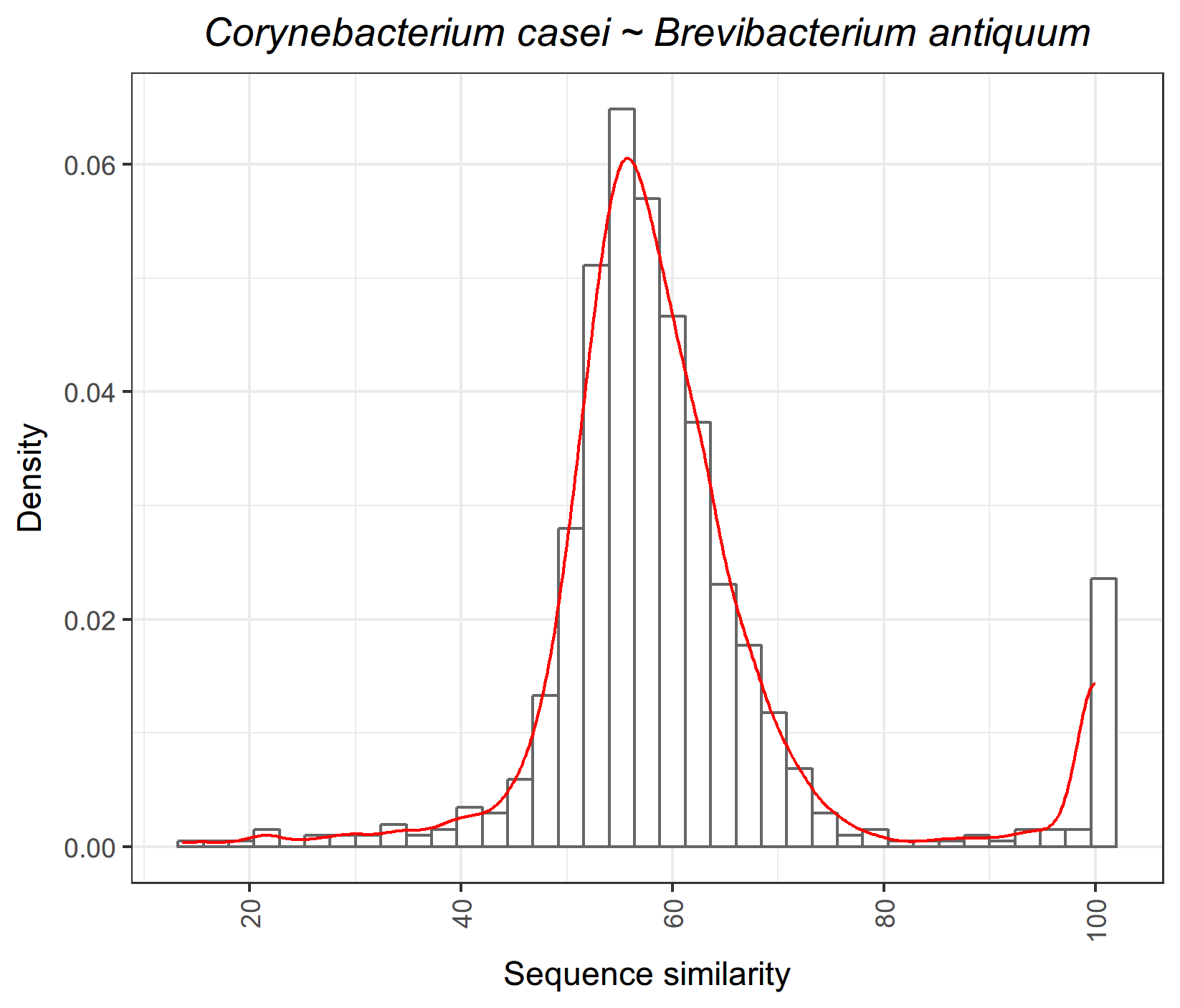

Supplement: Supplementary Figure S5 — Histogram for the sequence-similarity datasets of two cheese-associated bacteria, Brevibacterium antiquum CNRZ918 and Corynebacterium casei LMG S-19264. The red smoother line drawn over the histogram is the density curve. [file Image_5.TIF]
